# Supplementary material for: Generation of Comprehensive Ecosystem-Specific Reference Databases with Species-Level Resolution by High-Throughput Full-Length 16S rRNA Gene Sequencing and Automated Taxonomy Assignment (AutoTax)
Source: mBio. 2020 Sep 22;11(5):e01557-20. doi: 10.1128/mBio.01557-20 (PMC7512547; doi:10.1128/mBio.01557-20)
Supplement: TABLE S2 [file mBio.01557-20-st002.docx]

**Table S2: Samples used in this study.**

| Sample/SeqID | Environment | Location | Sampling_date | Latitude | Longitude | Design | PMID |
| --- | --- | --- | --- | --- | --- | --- | --- |
| MQ180605-111 | WWTP | Fredericia | 150824 | 55.552368 | 9.720404 | EBPR | This study |
| MQ180605-112 | WWTP | Randers | 150831 | 56.453767 | 10.070249 | EBPR | This study |
| MQ180605-113 | WWTP | Avedoere | 150902 | 55.607888 | 12.448949 | EBPR | This study |
| MQ180605-114 | WWTP | Damhusaaen | 150831 | 55.641184 | 12.505800 | EBPR | This study |
| MQ180605-115 | WWTP | Viborg | 150825 | 56.425251 | 9.455198 | EBPR | This study |
| MQ180605-116 | WWTP | Ribe | 150824 | 55.329053 | 8.74336 | EBPR | This study |
| MQ180605-117 | WWTP | Skive | 150825 | 56.565132 | 9.042158 | EBPR | This study |
| MQ180605-118 | WWTP | Ejby Moelle | 150825 | 55.399534 | 10.414787 | EBPR | This study |
| MQ180605-119 | WWTP | Esbjerg W | 150921 | 55.488097 | 8.430505 | BNR | This study |
| MQ180605-120 | WWTP | Aalborg E | 150824 | 57.045161 | 10.045761 | EBPR | This study |
| MQ180605-121 | WWTP | Haderslev | 150902 | 55.249786 | 9.508609 | EBPR | This study |
| MQ180605-122 | WWTP | Odense NE | 150908 | 55.432604 | 10.458855 | EBPR | This study |
| MQ180605-123 | WWTP | Odense NW | 150908 | 55.421534 | 10.366234 | BNR | This study |
| MQ180605-124 | WWTP | CP Kelco | 150901 | 55.507272 | 12.145488 | BNR | This study |
| MQ180605-125 | WWTP | Aalborg W | 150824 | 57.049513 | 9.864788 | EBPR | This study |
| MQ180605-126 | WWTP | Hjoerring | 150831 | 57.421265 | 9.975411 | EBPR | This study |
| MQ180605-127 | WWTP | Egaa | 150825 | 56.21314 | 10.242467 | EBPR | This study |
| MQ180605-128 | WWTP | Esbjerg E | 150826 | 55.458597 | 8.489092 | BNR | This study |
| MQ180605-129 | WWTP | Bjergmarken | 150825 | 55.649286 | 12.058568 | EBPR | This study |
| MQ180605-130 | WWTP | Boeslum | 150921 | 56.198467 | 10.728742 | EBPR | This study |
| MQ180605-131 | WWTP | Mariagerfjord | 150831 | 56.741769 | 10.115426 | EBPR | This study |
| MQ180605-132 | WWTP | Hirtshals | 150914 | 57.577275 | 9.992971 | EBPR | This study |
| 16SAMP-4598 | WWTP | Lundtofte | 140623 | 55.802079 | 12.538763 | EBPR | 28496434 |
| 16SAMP-4601 | WWTP | Ringkoebing | 140625 | 56.089793 | 8.236342 | EBPR | 28496434 |
| 16SAMP-2340 | WWTP | Aaby | 100819 | 56.150696 | 10.176274 | EBPR | 28496434 |
| 16SAMP-2643 | WWTP | Aars | 100819 | 56.810414 | 9.547714 | BNR | 28496434 |
| 16SAMP-2589 | WWTP | Fornaes | 110819 | 56.437583 | 10.913985 | BNR | 28496434 |
| MQ180605-133 | Anaerobic digester | Bjergmarken | 150826 | 55.6494 | 12.0589 | Thermophilic | This study |
| MQ180605-134 | Anaerobic digester | Ejby Moelle | 150826 | 55.3981 | 10.415 | Mesophilic | This study |
| MQ180605-135 | Anaerobic digester | Fredericia | 150826 | 55.5752 | 9.7476 | THP + Mesophilic | This study |
| MQ180605-136 | Anaerobic digester | Randers | 150826 | 56.4697 | 10.0373 | Mesophilic | This study |
| MQ180605-137 | Anaerobic digester | Slagelse | 150826 | 55.4163 | 11.3404 | Mesophilic | This study |
| MQ180605-138 | Anaerobic digester | Viborg | 150826 | 56.4251 | 9.4543 | Mesophilic | This study |
| MQ180605-139 | Anaerobic digester | Aaby | 150826 | 56.1512 | 10.1756 | Thermophilic | This study |
| MQ180605-140 | Anaerobic digester | Aalborg W | 150826 | 57.0493 | 9.8635 | Thermophilic | This study |
| MQ180605-141 | Anaerobic digester | Esbjerg W | 150826 | 55.4882 | 8.4307 | Mesophilic | This study |
| MQ180605-142 | Anaerobic digester | Damhusaaen | 150902 | 55.6408 | 12.5059 | Mesophilic | This study |
| MQ180605-143 | Anaerobic digester | Fornaes | 150902 | 56.4377 | 10.9145 | Mesophilic | This study |
| MQ180605-144 | Anaerobic digester | Hjoerring | 150902 | 57.4196 | 9.9756 | Mesophilic | This study |
| MQ180605-145 | Anaerobic digester | Avedoere | 150902 | 55.6087 | 12.4505 | Mesophilic | This study |
| MQ180605-146 | Anaerobic digester | Mariagerfjord | 150902 | 56.741769 | 10.115189 | Mesophilic | This study |
| MQ180605-147 | Anaerobic digester | Naestved | 150826 | 55.2111 | 11.7285 | THP + Mesophilic | This study |
| MQ180605-148 | Anaerobic digester | Soeholt | 150826 | 56.1753 | 9.5826 | Mesophilic | This study |
| 16SAMP-3460 | Anaerobic digester | Hobro | 130828 | 56.6375 | 9.8085 | Mesophilic | 28839166 |
| 16SAMP-3463 | Anaerobic digester | Lundtofte | 130828 | 55.8024 | 12.5388 | Mesophilic | 28839166 |
| 16SAMP-4077 | Anaerobic digester | Ringkoebing | 130828 | 56.0882 | 8.2568 | Mesophilic | 28839166 |
| 16SAMP-4081 | Anaerobic digester | Egaa | 130501 | 56.21314 | 10.242467 | Mesophilic | This study |
| 16SAMP-3459 | Anaerobic digester | Hobro | 130821 | 56.1353 | 8.9731 | NA | This study |
| 16SAMP-4076 | Anaerobic digester | Nysted | 130821 | 54.699063 | 11.712259 | NA | This study |
| * THP: thermal hydrolysis pretreatment; PMID: Pubmed ID reference | | | |  |  |  |  |
