# Supplementary material for: Generation of Comprehensive Ecosystem-Specific Reference Databases with Species-Level Resolution by High-Throughput Full-Length 16S rRNA Gene Sequencing and Automated Taxonomy Assignment (AutoTax)
Source: mBio. 2020 Sep 22;11(5):e01557-20. doi: 10.1128/mBio.01557-20 (PMC7512547; doi:10.1128/mBio.01557-20)
Supplement: TEXT S1 [file mBio.01557-20-s0001.docx]

SUPPLEMENTARY INFORMATION FOR

Generation of comprehensive ecosystems-specific reference databases with species-level resolution by high-throughput full-length 16S rRNA gene sequencing and automated taxonomy assignment (AutoTax)

Morten Simonsen Dueholm, Kasper Skytte Andersen, Simon Jon McIlroy, Jannie Munk Kristensen, Erika Yashiro, Søren Michael Karst, Mads Albertsen, and Per Halkjær Nielsen

Center for Microbial Communities, Department of Chemistry and Bioscience, Aalborg University, Aalborg, Denmark.

**Detailed description of the AutoTax procedure:**

*Full-length 16S rRNA exact amplicon sequence variant (FL-ASV) calling*

Full-length 16S rRNA gene sequences are first oriented according to the SILVA 138 SSURef Nr99 database (1) using the *usearch -orient* command. The sequences are then dereplicated using *usearch -fastx_uniques* with the *-sizeout, -strand plus*, and *-threads 1* options. The *-sizeout* option put a size annotation (how many times a unique sequence was observed) in the FASTA header. The *-strand plus* option ensures that only correctly oriented sequences are considered when identifying identical sequences. The dereplicated sequences are sorted and numbered based on the size annotation. The *-threads 1* option ensures that sequences with the same size are always sorted and numbered the same way in the output. The dereplicated sequences are finally denoised to produce the FL-ASVs using the *usearch -unoise3* command with the *-minsize 2* option. The *-minsize* options specify the minimum abundance required for detecting an ASV. The default value for short-read amplicons is 8; however as the synthetic long-read sequences are independently amplified (due to the unique molecular identifiers (UMIs) added before the PCR steps) and have a very low error-rate we can lower the threshold to 2 with little risk of introducing sequences with errors, see "Calculation of FL-ASV error-rate" below.

*Preparation of chimera-filtered full-length 16S rRNA OTUs (FL-OTUs)*

Dereplicated sequences from above are clustered at 99% sequence identity to create FL-OTUs using *usearch -cluster_smallmem* command with the *-id 0.99, -maxrejects 0, -centroids,* and *-sortedby size* options. The *-maxrejects 0* option specified a complete database search, thereby providing more confident clusters. Potential chimeras are identified and extracted using the *usearch -uchime2_ref* command with the *-strand plus, -mode sensitive*, and -chimeras options using the FL-ASVs as a reference database. The sensitive mode was chosen as it captures more chimeras (on the expense of more false positives), as these are detrimental to the final reference database. The FL-ASVs were used as the reference database because it contains exact references for the abundant 16S rRNA genes, which are most likely to form chimeras. Chimeras are finally removed to create the chimera-filtered FL-OTUs using the *usearch -search_exact* command with the *-strand plus -dbnotmatched* options, using the identified chimera sequences as the query, and the pre-filtered FL-OTUs as the reference database.

*Taxonomy assignment*

For the taxonomy assignment, we first create two independent taxonomies. The first taxonomy is based on the most recent version of the SILVA SSURef Nr99 database and reflects the current state of microbial taxonomy. The second is a robust, although not necessarily evolutionary correct, *de novo* taxonomy. The latter will be used as a taxonomic placeholder for taxonomic ranks without information in the SILVA-based taxonomy.

The first step in both cases is the alignment of each FL-ASV to the global alignment in the SILVA reference database in ARB-format using SINA. The aligned sequences are hereafter trimmed to position 1048 to 41788 in the global SILVA alignment using the generic Linux command awk. The purpose of this trimming is two-fold. Firstly, it ensures that flanking sequences do not result in artificially low identities when the FL-ASVs are mapped to reference sequences in the SILVA database produced with the popular 27F and 1391R primers (2). Secondly, it allows a more robust *de novo* clustering (3). After trimming, gaps are removed from the alignment of FL-ASVs using the *usearch -fasta_stripgaps* command. Finally, the FL-ASVs are sorted based on the FL-ASV numbers in R.

The SILVA based taxonomy is obtained by mapping each of the trimmed FL-ASVs to the most recent version of the SILVA SSURef Nr99 and the type strain database in FASTA format and obtaining the taxonomic information from the closest relative as well as the percent identity. Mappings are done using the *usearch -usearch_global* command with the *-maxrejects 0, -maxaccepts 0, -top_hit_only, -strand plus, -id 0,* and *-blast6out* options for the complete SILVA database and the *-maxrejects 0, -maxaccepts 0, -strand plus, -id 0.987,* and *-blast6out* for the type strains. The *-maxrejects 0* and  *-maxaccepts 0* options ensures that a comprehensive search is performed. The *-top_hits_only* and *-id 0* options provide the best hit in the complete SILVA database, whereas the *-id 0.987* without the *-top_hit_only* option provides all hits within the species-level threshold. The output files from the SILVA reference mapping are loaded into R, and a data frame with columns for FL-ASV number, percent identity, and SILVA taxonomy of the closest relative is created. The taxonomy field is then split into the seven main taxonomic ranks from kingdom to species. Fields that contain any of the following whole words are cleared: "uncultured", "unknown", "unidentified", "incertae sedis", "metagenome", "bacterium", and "possible". In addition, we replace all "candidatus" with "Ca" and all white spaces with underscores. Finally, all characters except letters, numbers and period, dash, and underscore are removed.

The taxonomy obtained from the closest relative in the SILVA database does not necessarily match that of the FL-ASV. The taxonomy is therefore trimmed based on the percent identity between the FL-ASV and its closest relative. For this trimming we use the taxonomic thresholds proposed by Yarza *et al.* (4) (94.5% for genus-level, 86.5% for family-level, 82.0%, for order-level, 78.5% for class-level, and 75.0% for phylum-level). Species-level classifications are only obtained from the mapping against type strains, and no classifications are provided if reference sequences from more than one species are within the species-level threshold.

To generate a comprehensive *de novo* taxonomy, FL-ASVs are clustered based on the taxonomic thresholds proposed by Yarza *et al.* (4) using the *usearch -cluster_smallmem* command with the *-id x, -maxrejects 0, -uc,* and *-sortedby other* options. Where x represent the threshold for the given taxonomic rank. The cluster_smallmem algorithm with the *-sortedby other* option clusters the FL-ASVs based on when they appear in the input FASTA file, and the same clusters are therefore formed even though additional FL-ASVs are appended to the FL-ASV-database in the future. We confirmed this by processing only the first half of the FL-ASVs from this study, which provided identical clustering. The *-maxrejects 0* ensures that the best reference is found among all clustered sequences. The output file is a UCLUST-format tabbed text, which describes the clusters formed.

The six UCLUST output files (species- to phylum-level) are loaded into R, and each are converted into a data frame with two columns. The first column with clusters is named based on the taxonomic clustering rank, and the second column with the input sequences is named as the taxonomic rank below. Subsequently, the data frames are merged from species to phylum level. This results in a comprehensive taxonomy, where the clustered centroids determine affiliation to the taxonomic ranks above.

The SILVA-based taxonomy and the *de novo* taxonomy are finally merged in R by replacing empty fields in the SILVA-based taxonomy with the *de novo* taxonomic information. This results in a comprehensive taxonomy, where all FL-ASVs are described on all seven taxonomic ranks.

Merging of the two taxonomies results in a few cases where a taxon has more than one parent (e.g., sequences from the same species affiliate more than one genus). In these cases, the taxonomy of the FL-ASV with the lowest ASV-number within the taxa is assigned to all members. A log file is written with details of all such cases.

**Calculation of FL-ASV error-rate:**

The Unoise3 algorithm predicts which sequences represent true biological variants (ASVs) based on their relative abundance compared to those of closely related sequences with one or a few errors. Based on the error-rate profile of full-length 16S rRNA genes found in Karst et al. (5) and the assumption that sequencing-errors are randomly distributed we can calculate the relative chance of obtaining a correct ASV compared to an ASV with a single sequencing error when we only consider sequence seen at least twice:

The probability of obtaining a correct sequence for FL-ASV_n_ is the product of the probability of obtaining a sequence without any errors and relative abundance of FL-ASV_n_, and for the sequence to be seen twice, we need to square this number.

$$P_{correct}\left( {FL.ASV}_{n} \right)=\left( P_{no error}\times P_{{FL.ESV}_{n}} \right)^{2}$$

The probability of obtaining a sequence with a single error for FL-ASV_n_ is the product of the probability of obtaining a sequence with one error and relative abundance of FL-ASV_n_. However, to calculate the probability of picking a sequence with the same error, we need to divide this probability with three times the length of FL-ASV_n_ because the error could occur at any position in the sequence and could be any of the three wrong nucleotides. The probability for seeing a specific incorrect sequence twice therefore becomes:

$$P_{one error}\left( {FL.ASV}_{n} \right)=P_{one error}\times P_{FL.{ASV}_{n}}\times\frac{P_{one error}\times P_{{FL.ASV}_{n}}}{3\times{length}_{{FL.ASV}_{n}}(bp)}$$

The relative chance of obtaining a correct FL-ASV compared to an FL-ASV with a single sequencing error is therefore:

$$\frac{P_{correct}\left( {FL.ASV}_{n} \right)}{P_{one error}\left( {FL.ASV}_{n} \right)}=\frac{{P_{no error}}^{2}\times3\times{length}_{{FL.ASV}_{n}}(bp)}{{P_{one error}}^{2}}=\frac{{0.112}^{2}\times3\times1450}{{0.209}^{2}}=1249$$

The results show that for each time we obtain an FL-ASV with a single error, we should expect approximately 1250 times more sequences of the correct FL-ASV. The difference should be large enough for Unoise3 to be able to remove almost all sequences with errors even though the errors are not entirely randomly distributed.

To confirm this, we reanalyzed the mock data used in Karst *et al.* (5). This data contained 7816 trimmed sequences (27f to 1492r) from a balanced mixture of DNA from *Escherichia coli* MG1655 (NC_000913), *Bacillus subtilis* subsp. subtilis str. 168 (NC_000964) and *P. aeruginosa* PAO1 (NC_002516). Denoising with *usearch -unoise3* command and the *-minsize 2* option produced 16 error-corrected sequences, of which 13 mapped perfectly to the 16S rRNA reference sequences, two mapped with a single error, and one mapped with five errors. As the two FL-ASVs with single errors were observed 14 and 4 times, respectively, it is highly unlikely that they represent sequencing error based on the previous error-rate calculations. The same is holds for the FL-ASV with five errors, although this sequence was observed only twice.

**References:**

1. Quast C, Pruesse E, Yilmaz P, Gerken J, Schweer T, Yarza P, Peplies J, Glöckner FO. 2013. The SILVA ribosomal RNA gene database project: Improved data processing and web-based tools. Nucleic Acids Res 41:D590-6.

2. Klindworth A, Pruesse E, Schweer T, Peplies J, Quast C, Horn M, Glöckner FO. 2013. Evaluation of general 16S ribosomal RNA gene PCR primers for classical and next-generation sequencing-based diversity studies. Nucleic Acids Res 41:1–11.

3. Schloss PD. 2013. Secondary structure improves OTU assignments of 16S rRNA gene sequences. ISME J 7:457–460.

4. Yarza P, Yilmaz P, Pruesse E, Glöckner FO, Ludwig W, Schleifer K-H, Whitman WB, Euzéby J, Amann R, Rosselló-Móra R. 2014. Uniting the classification of cultured and uncultured bacteria and archaea using 16S rRNA gene sequences. Nat Rev Microbiol 12:635–645.

5. Karst SM, Dueholm MS, McIlroy SJ, Kirkegaard RH, Nielsen PH, Albertsen M. 2018. Retrieval of a million high-quality, full-length microbial 16S and 18S rRNA gene sequences without primer bias. Nat Biotechnol 36:190–195.
