# Supplementary figures and images for: Generation of Comprehensive Ecosystem-Specific Reference Databases with Species-Level Resolution by High-Throughput Full-Length 16S rRNA Gene Sequencing and Automated Taxonomy Assignment (AutoTax)
Source: mBio. 2020 Sep 22;11(5):e01557-20. doi: 10.1128/mBio.01557-20 (PMC7512547; doi:10.1128/mBio.01557-20)

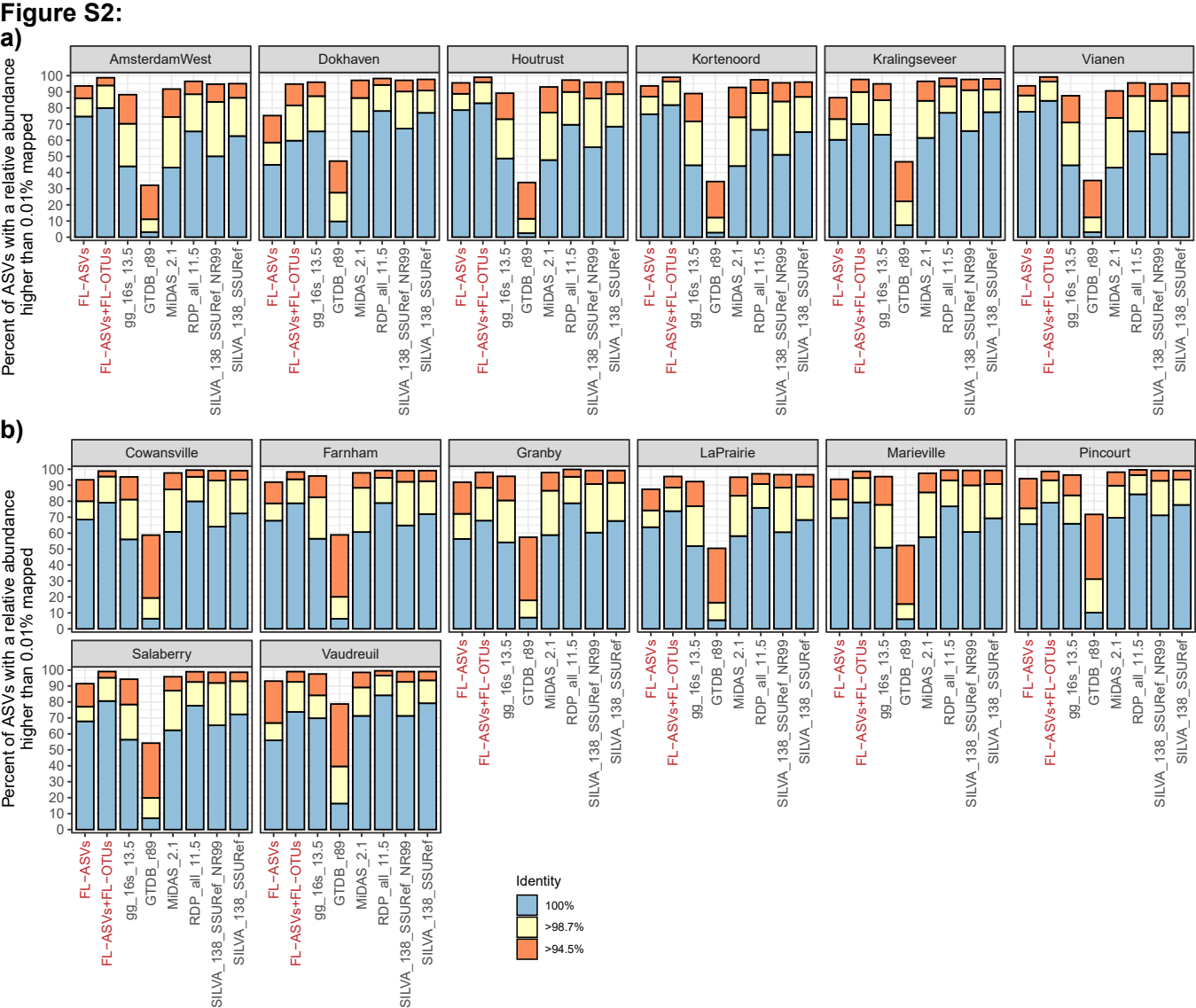

Supplement: FIG S2 [file mBio.01557-20-sf002.pdf]

**Figure S1:**

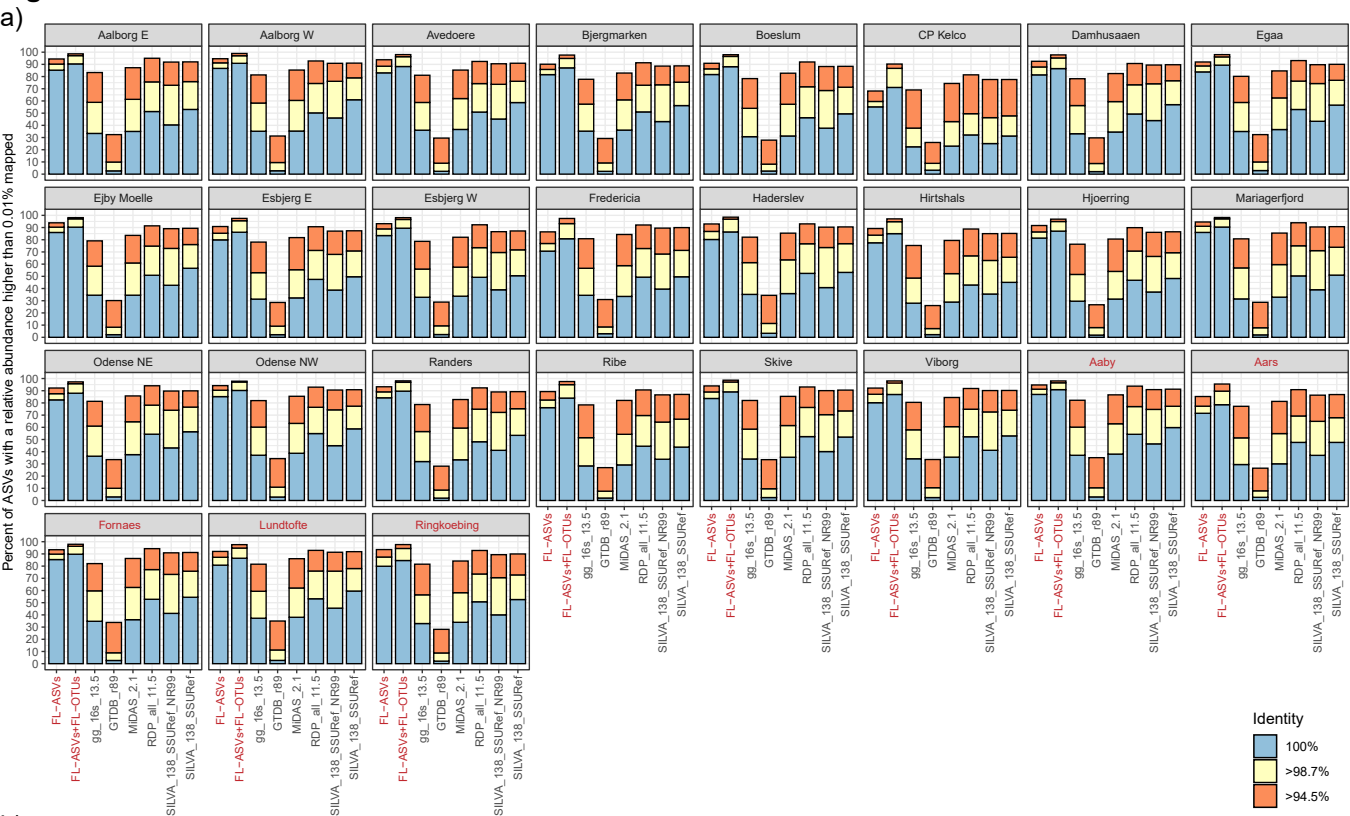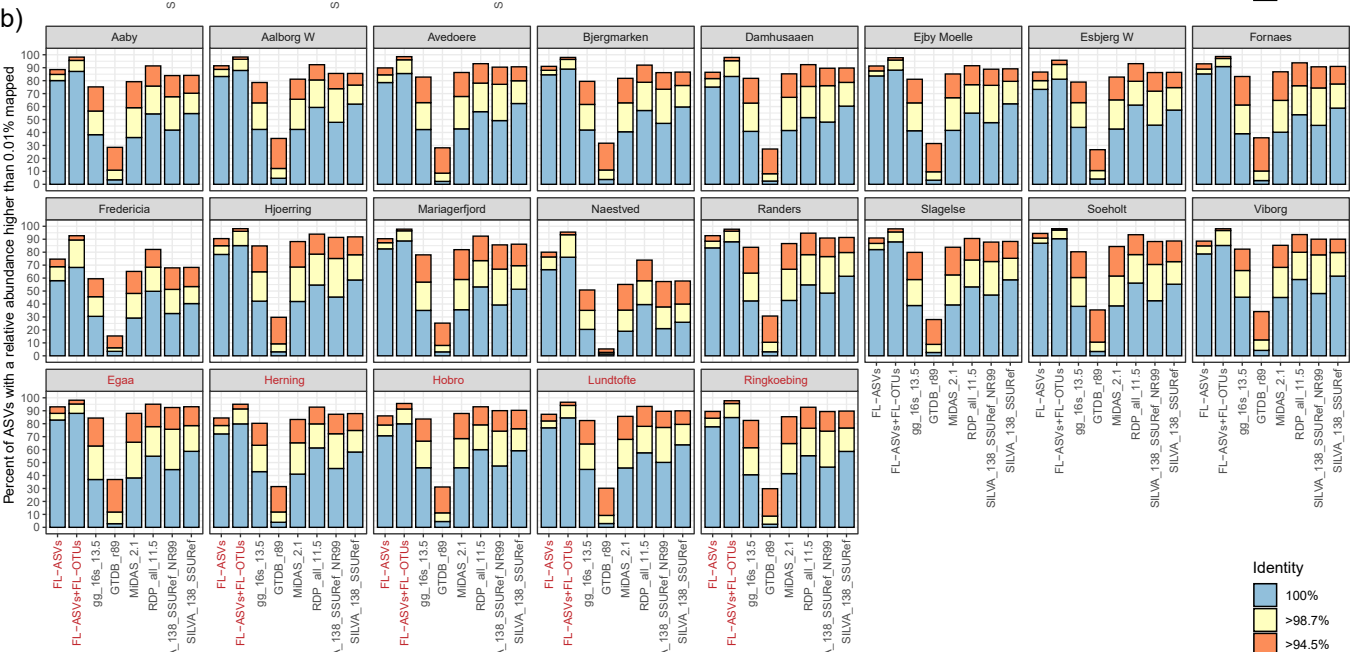

Supplement: FIG S1 [file mBio.01557-20-sf001.pdf]

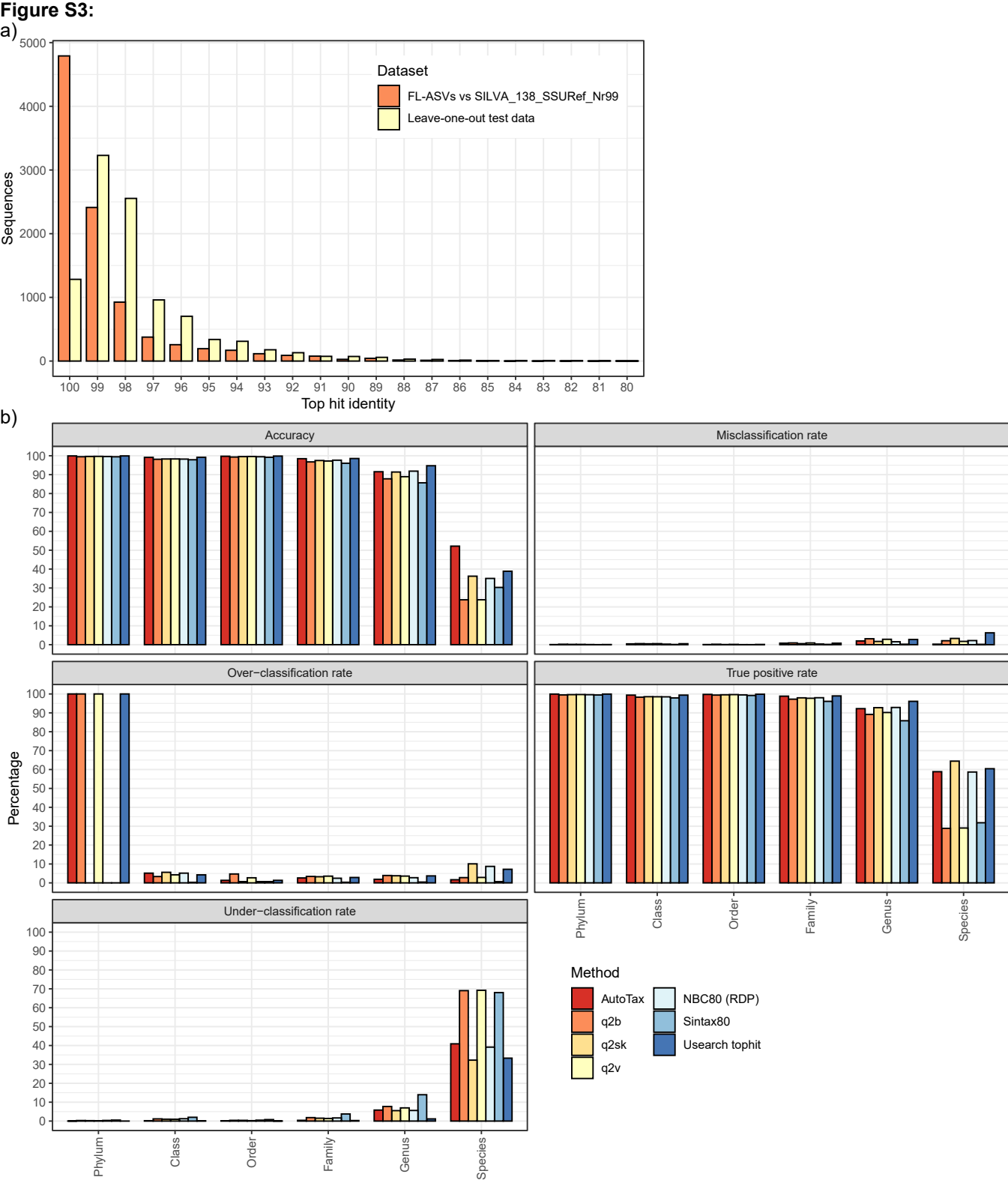

Supplement: FIG S3 [file mBio.01557-20-sf003.pdf]

Number of taxa that are assigned denovo names

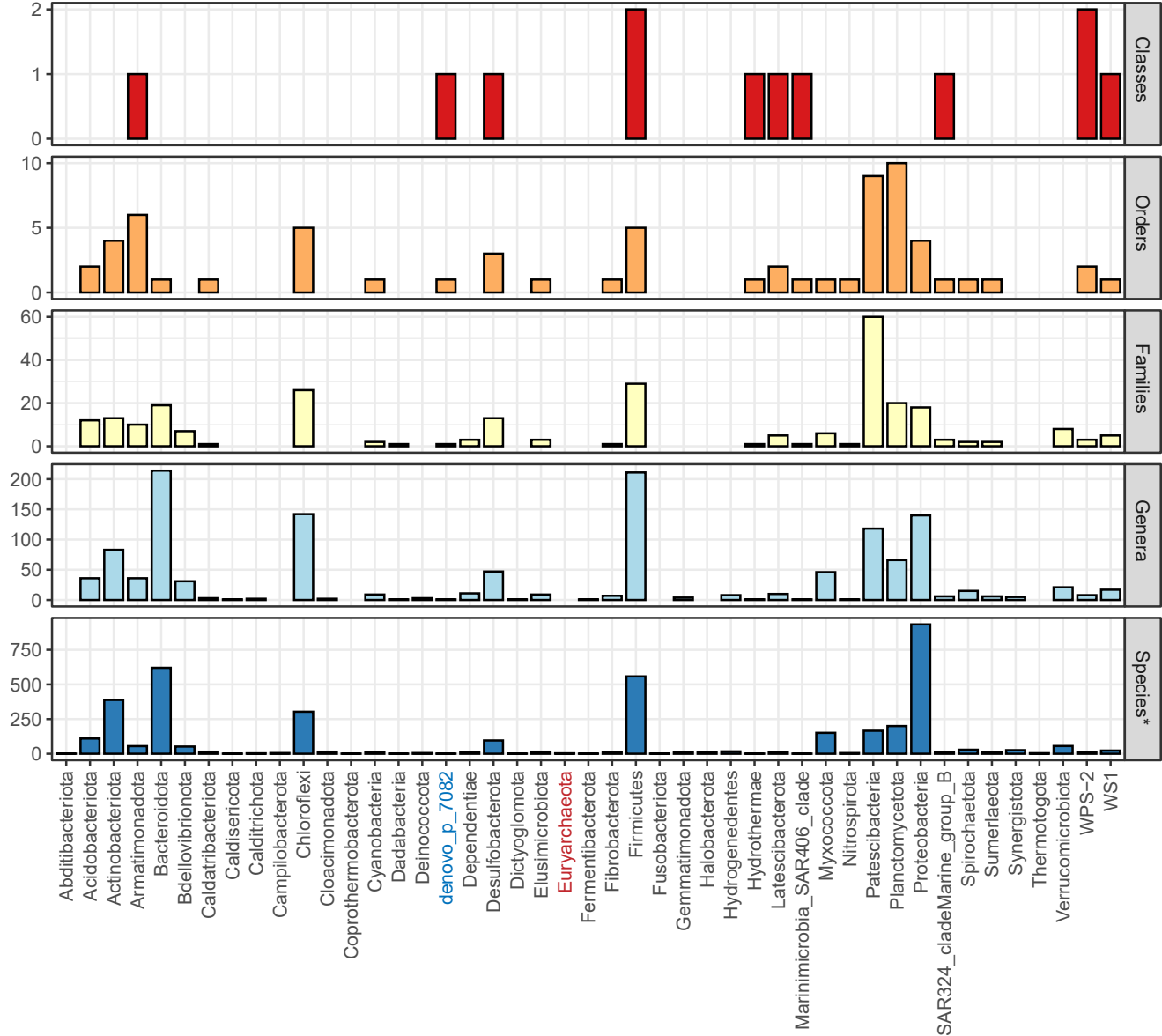

Supplement: FIG S4 [file mBio.01557-20-sf004.pdf]

Figure S5:

Percent of ASVs with a relative abundance higher than 0.001% classified

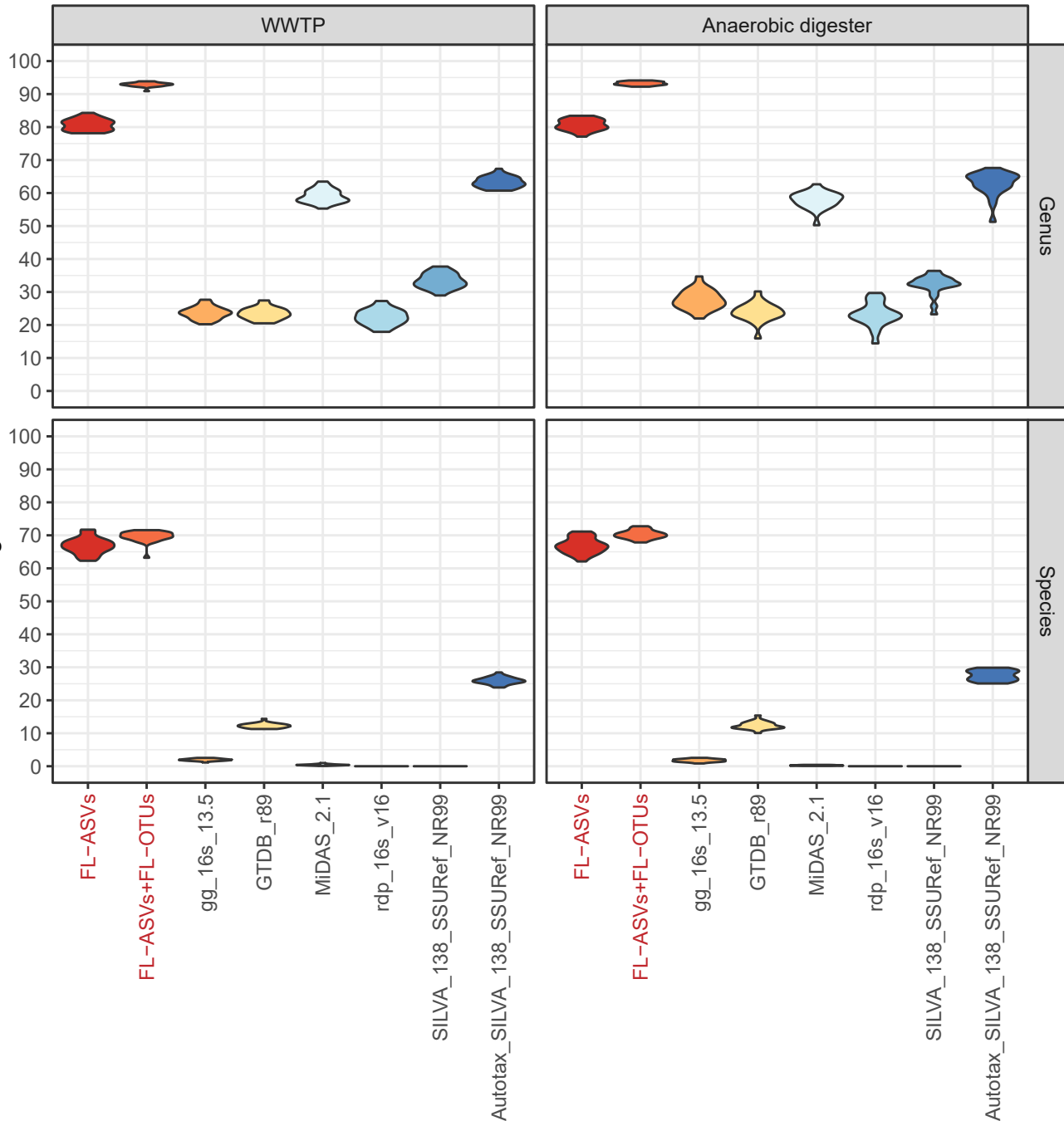

Supplement: FIG S5 [file mBio.01557-20-sf005.pdf]

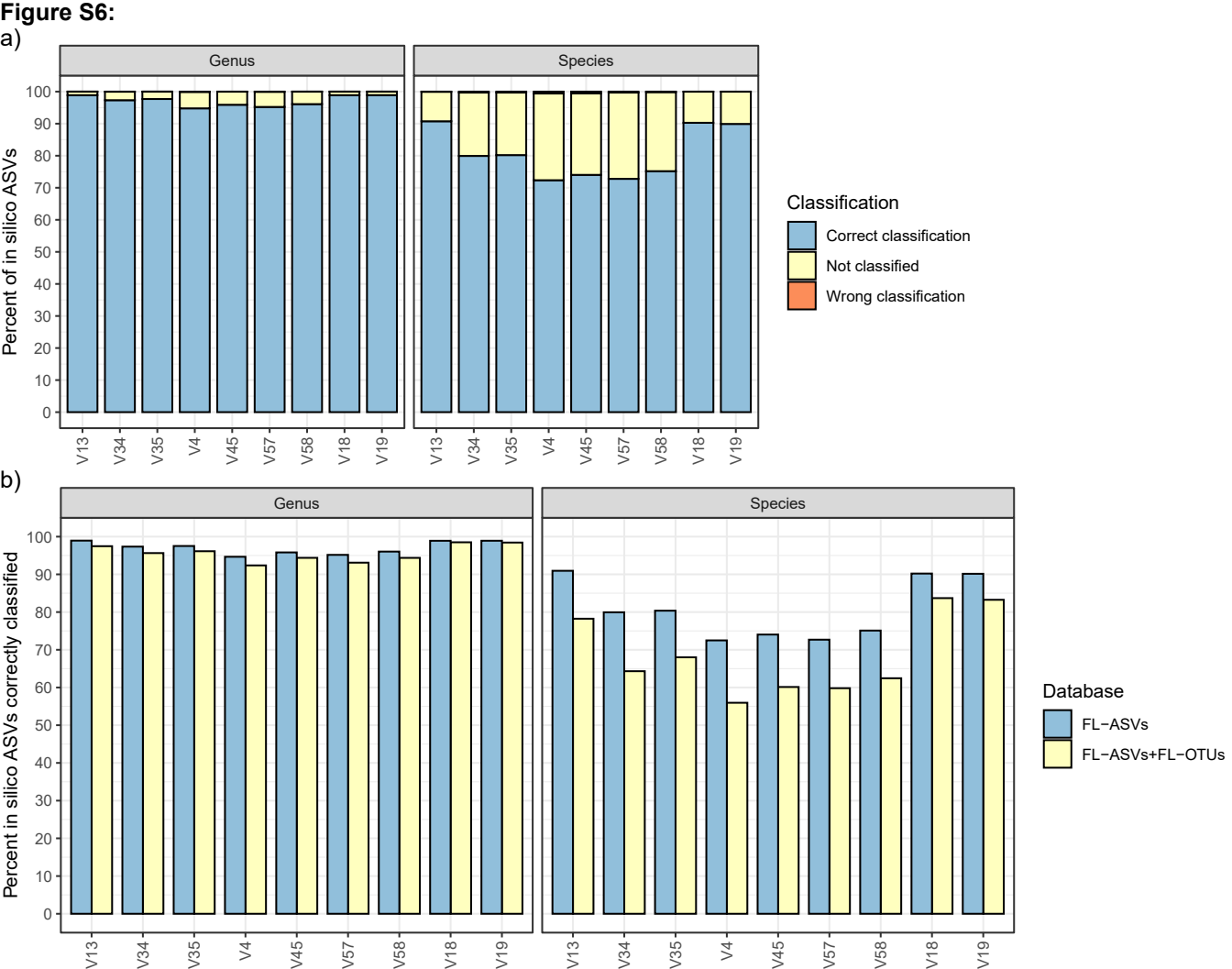

Supplement: FIG S6 [file mBio.01557-20-sf006.pdf]
