# Supplementary material for: Generation of Comprehensive Ecosystem-Specific Reference Databases with Species-Level Resolution by High-Throughput Full-Length 16S rRNA Gene Sequencing and Automated Taxonomy Assignment (AutoTax)
Source: mBio. 2020 Sep 22;11(5):e01557-20. doi: 10.1128/mBio.01557-20 (PMC7512547; doi:10.1128/mBio.01557-20)
Supplement: TABLE S1 [file mBio.01557-20-st001.docx]

**Table S1: Oligonucleotides used for full-length 16S rRNA gene library preparation.** Unique molecular tags, and sample barcodes are marked with blue and red, respectively. The GGGGGG motif marked with bold typefaces may form strong secondary structures known as guanine tetraplexes. This motif was replaced by GCGCGG in the DNA-based protocol, which increased the robustness of library preparation. Oligoes marked with “*” are identical between the RNA- and DNA-based protocols.

| **Name** | **Sequence** | **Protocol step** |
| --- | --- | --- |
| SSU_rRNA_RT2 | A**GGGGGG**CAAAGATGAAGATNNNNNNNNNNNNNNNCTAGTACGACTTGCCTGTCGCTCTATCTTCTTTTTTTTTTTTTTTTTTTTVN | First strand cDNA synthesis |
| SSU_rRNA_RT3 | A**GGGGGG**CAAAGATGAAGATNNNNNNNNNNNNNNNTTCTGCCTACTTGCCTGTCGCTCTATCTTCTTTTTTTTTTTTTTTTTTTTVN |  |
| SSU_rRNA_s | 5'Phos-GGGCAATATCAGCACCAACAGAAA-3'SpC3 | Adaptor ligation |
| SSU_rRNA_l | CTCCACCCAGACTCATCCATNNNNNNNNNNNNNNNATAGAGGCTTTCTGTTGGTGCTGATATTGC | Second strand cDNA syntehsis |
| SSU_rRNA_pcr_fw* | CTCCACCCAGACTCATCCAT | Library and clonal amplification |
| SSU_rRNA_pcr_rv | A**GGGGGG**CAAAGATGAAGAT |  |
| SSU_rRNA_readtag_fw* | CAAGCAGAAGACGGCATACGAGATGTGACTGGAGTTCAGACGTGTGCTCTTCCGATCTCTCCACCCAGACTCATCCAT | Read-tag library preparation |
| SSU_rRNA_readtag_rv | CAAGCAGAAGACGGCATACGAGATGTGACTGGAGTTCAGACGTGTGCTCTTCCGATCTA**GGGGGG**CAAAGATGAAGAT |  |
| SSU_rRNA_linktag_fw | AATGATACGGCGACCACCGAGATCT ACACTCTTTCCCTACACGACGCTCTTCCGATCTGAAGATAGAGCGACAGGCAAGT | Linked-tag library preparation |
| SSU_rRNA_linktag_rv | CAAGCAGAAGACGGCATACGAGATCGGT CTCGGCATTCCTGCTGAACCGCTCTTCCGATCTGCAATATCAGCACCAACAGAAA |  |
| SSU_rRNA_read2_fw* | GCTCTTCCGATCTCTCCACCCAGACTCATCCAT | Illumina MiSeq/HiSeq sequencing |
| SSU_rRNA_read2_rv | GCTCTTCCGATCTA**GGGGGG**CAAAGATGAAGAT |  |
| f16S_rDNA_pcr1_fw1 | CTCCACCCAGACTCATCCATNNNNNNNNNNNNNNNTGCCTCTTAGAGTTTGATCMTGGCTCAG | Adaptor tagging |
| f16S_rDNA_pcr1_fw2 | CTCCACCCAGACTCATCCATNNNNNNNNNNNNNNNTCCTCTACAGAGTTTGATCMTGGCTCAG |  |
| f16S_rDNA_pcr1_rw-v2 | A**GCGCGG**CAAAGATGAAGATNNNNNNNNNNNNNNNCGTACTAGTACGGYTACCTTGTTACGACTT |  |
| f16S_rDNA_pcr2_fw* | CTCCACCCAGACTCATCCAT | Library and clonal amplification |
| f16S_rDNA_pcr2_rv-v2 | A**GCGCGG**CAAAGATGAAGAT |  |
| f16S_rDNA_readtag_fw* | CAAGCAGAAGACGGCATACGAGAT GTGACTGGAGTTCAGACGTGTGCTCTTCCGATCTCTCCACCCAGACTCATCCAT | Read-tag library preparation |
| f16S_rDNA_readtag_rv | CAAGCAGAAGACGGCATACGAGATGTGACTGGAGTTCAGACGTGTGCTCTTCCGATCTA**GCGCGG**CAAAGATGAAGAT |  |
| f16S_rDNA_linktag_fw | CAAGCAGAAGACGGCATACGAGATCGGTCTCGGCATTCCTGCTGAACCGCTCTTCCGATCTCTGAGCCAKGATCAAACTCT | Linked-tag library preparation |
| f16S_rDNA_linktag_rv | AATGATACGGCGACCACCGAGATCTACACTCTTTCCCTACACGACGCTCTTCCGATCTAAGTCGTAACAAGGTARCCGTA |  |
| f16S_rDNA_read2_fw* | GCTCTTCCGATCTCTCCACCCAGACTCATCCAT | Illumina MiSeq/HiSeq sequencing |
| f16S_rDNA_read2_rv-v2 | GCTCTTCCGATCTA**GCGCGGC**AAAGATGAAGAT |  |
