# Supplementary material for: Generation of Comprehensive Ecosystem-Specific Reference Databases with Species-Level Resolution by High-Throughput Full-Length 16S rRNA Gene Sequencing and Automated Taxonomy Assignment (AutoTax)
Source: mBio. 2020 Sep 22;11(5):e01557-20. doi: 10.1128/mBio.01557-20 (PMC7512547; doi:10.1128/mBio.01557-20)
Supplement: TABLE S3 [file mBio.01557-20-st003.docx]

**Table S3: Classification of raw assemblies, full-length 16S rRNA sequences, and FL-ASVs**

| **Environment** | **Library type** | **Data set** | **nSeqs** | **A16S** | **A23S** | **B16S** | **B23S** | **E18S** | **E28S** | **Unclassified** |
| --- | --- | --- | --- | --- | --- | --- | --- | --- | --- | --- |
| Activated sludge | RNA based | Raw assemblies | 274,240 | 2,730 | 306 | 119,947 | 129,851 | 6,139 | 13,689 | 1,540 |
| Activated sludge | RNA based | Full-length 16S rRNA | 45,907 | 396 | 0 | 45,496 | 0 | 15 | 0 | 0 |
| Activated sludge | DNA based | Raw assemblies | 506,519 | 0 | 0 | 506,518 | 0 | 0 | 0 | 1 |
| Activated sludge | DNA based | Full-length 16S rRNA | 468,426 | 0 | 0 | 468,425 | 0 | 0 | 0 | 1 |
| Anaerobic digester | RNA based | Raw assemblies | 361,091 | 73,583 | 8,990 | 79,057 | 196,916 | 0 | 206 | 2,339 |
| Anaerobic digester | RNA based | Full-length 16S rRNA | 38,834 | 16,494 | 0 | 22,340 | 0 | 0 | 0 | 0 |
| Anaerobic digester | DNA based | Raw assemblies | 402,232 | 0 | 0 | 402,232 | 0 | 0 | 0 | 0 |
| Anaerobic digester | DNA based | Full-length 16S rRNA | 373,340 | 0 | 0 | 373,340 | 0 | 0 | 0 | 0 |
| Combined | Combined | FL-ASVs | 9,521 | 75 | 0 | 9,446 | 0 | 0 | 0 | 0 |
